# Fine tuning for totally endoscopic mitral valve surgery: ERAS applications

**DOI:** 10.3389/fcvm.2024.1398438

**Published:** 2024-10-10

**Authors:** Serkan Ertugay, Sedat Karaca, Ayşen Yaprak Engin, Ümit Kahraman, Zehra Ünlü, Seden Kocabaş, Tanzer Çalkavur, Mustafa Özbaran

**Affiliations:** ^1^ Department of Cardiovascular Surgery, Ege University Faculty of Medicine, Izmir, Türkiye; ^2^ Department of Anesthesiology, Ege University Faculty of Medicine, Izmir, Türkiye

**Keywords:** mitral valve surgery, enhanced recovery, patient blood management (PBM), minimally invasive cardiac surgery, endoscopic surgery

## Abstract

**Aim:**

One of the philosophies of minimally invasive mitral surgery is to enhance recovery after surgery (ERAS). Beyond surgical applications, ERAS applications provide a complementary approach to optimize postoperative course and discharge. In this report, we aim to present institutional protocol for ERAS and its results in patients who underwent totally endoscopic mitral valve surgery (TEMVS).

**Patients and methods:**

Between 2021 and 2023, totally 113 patients who underwent TEMVS were included in this study. TEMVS was performed by peripheral cannulation and 3D endoscopic technique. As a dedicated team, institutional ERAS protocols which are used are listed above: (1) Education; operative course, cessation of smoking and alcohol. (2) Anemia; diagnostic evaluation and its treatment by iv iron. (3) Optimization of blood glucose; checking of HbA1c and control of hyperglycemia. (4) Rehabilitation; Physical and pulmonary rehabilitation. (5) Anxiety and Analgesia treatment. (6) Blood Conservation techniques; Antifibrinolytic, acute normovolemic hemodilution, less priming volume, mini-incision, meticulous surgery by 3D endoscope. (7) Postoperative; early extubation, prevention of nausea, aggressive analgesia, early mobilization, early removal of tubes. (8) Restrictive transfusion strategy. (9) Early discharge.

**Results:**

The mean age was 54.7 years, and 56% was female. The rate of iv iron therapy for anemia was 26.5%. Mitral repair was performed in 58.4% of the cases. The repair rate of degenerative mitral valve was 96.9%. Of all, 68.1% did not have any red packed cells and 15.9% had only one unit. Ninety-five patients (90.2%) did not have any unit of fresh frozen plasma. The median extubation time was 7 h. On the postoperative first day, 96% of foley catheters, 87% of all central venous catheter and 93% of all drainage tubes are removed. The rates of respiratory, infectious, and renal complications were 9%, 3.5%, 3.4% respectively. The median ICU, and hospital stays were 1 and 5 days respectively. There was only one mortality in the early postoperative period.

**Conclusion:**

Totally endoscopic mitral valve surgery provides minimal surgical trauma. By the addition of well-established and nurse-based ERAS protocols, complication and transfusion rates can be decreased, early recovery and discharge can be provided.

## Introduction

Totally endoscopic mitral valve surgery (TEMVS) offers one of the least invasive surgical technique for mitral valve therapies (1). This method is distinguished by the utilization of peripheral cannulation and state-of-the-art 3D endoscopic technology. By the totally endoscopic approach, large incisions or rib spreading retractor are not needed, thereby complications such as bleeding and pain are avoided. However, the full potential of TEMVS can only be realized when complemented by effective perioperative care strategies, underscoring the importance of Enhanced Recovery After Surgery (ERAS) protocols in this context (2).

ERAS is a patient-centered, multidisciplinary approach that encompasses a series of preoperative, intraoperative, and postoperative strategies aimed at reducing surgical trauma, optimizing postoperative recovery and early discharge (3). The integration of ERAS into mitral valve surgery not only has the potential to improve patient outcomes but is also in line with the evolving healthcare landscape that emphasizes patient wellbeing and resource optimization. Although, a new guideline aiming to represent the latest evidence, is published by the ERAS Society, the care elements of the ERAS programs may differ based on institutional dynamics, possibilities, and experiences (4).

This study aims to present an institutional algorithm for ERAS applications and its clinical outcomes in patients who underwent totally endoscopic mitral valve surgery.

## Materials and methods

This is a cohort study that was retrospectively designed and conducted at the Department of Cardiovascular Surgery in Ege University School of Medicine. Approval for this study was obtained from the local ethical committee with the document number E-99166796-050.04-1738888. From 2021 to January 2024, a total of 117 patients who provided informed consent, underwent mitral valve surgery in our institution as part of the Totally Endoscopic (3D, Aesculap AG, Tuttlingen, Germany) Mitral Surgery program. During the learning curve period, patients were carefully selected for TEMVS, excluding those with aortic regurgitation more than mild, a history of previous thoracotomy, severe mitral annular calcification, and severe peripheral arterial disease. Four patients who required conversion to sternotomy due to unpredicted lung adhesions and cannulation complications were excluded from the data analysis.

### ERAS program

**Figure 1:**
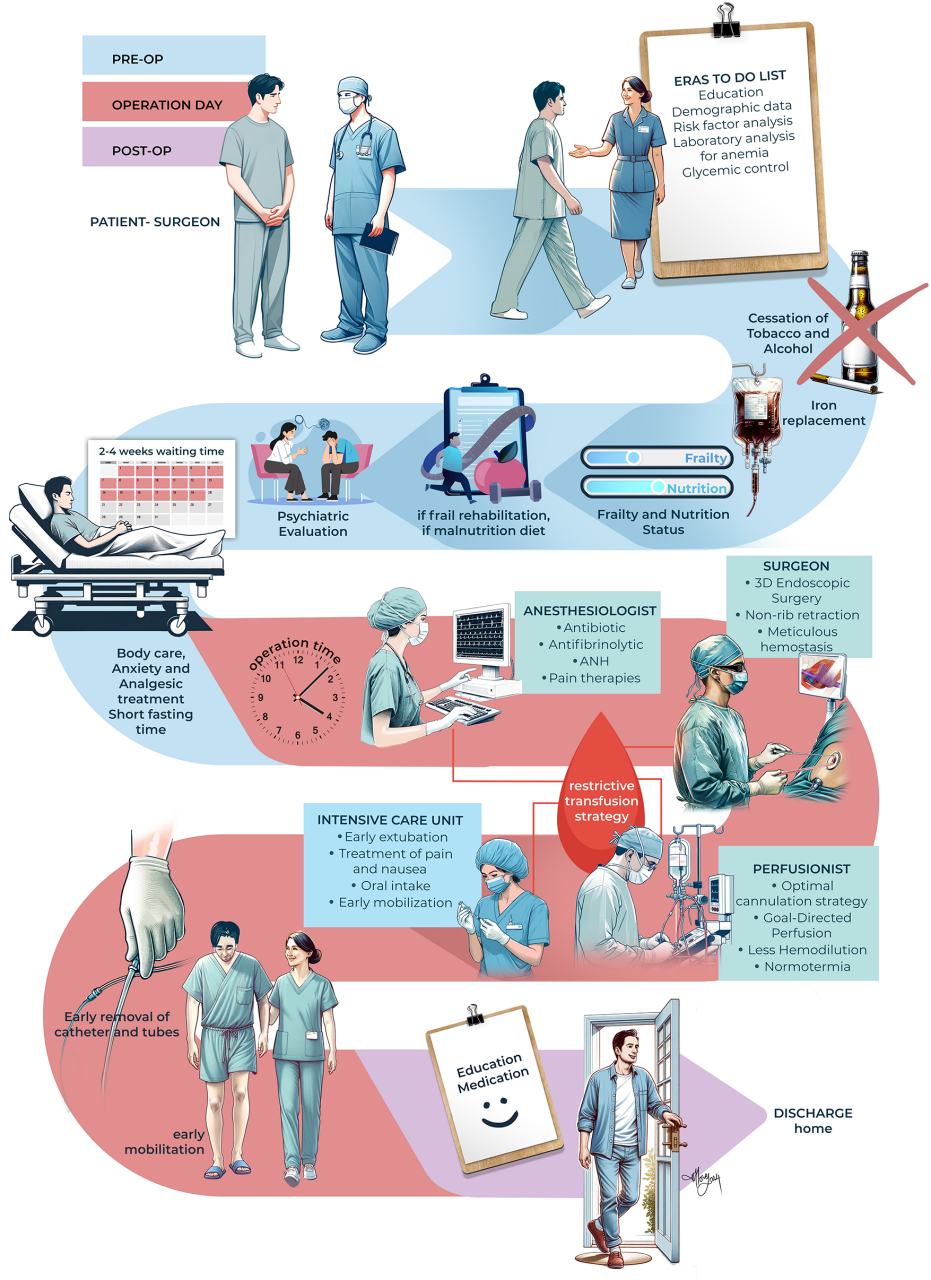
Institutional protocol for ERAS starting from the decision of surgery until discharge. (ANH, acute normovolemic hemodilution). Illustrated by Merve Evren (2024).

While the surgeon leads this program, the nursing staff are the main stakeholders. The team consists of three nurses who are primarily responsible for the preoperative ERAS clinic, intensive care unit (ICU), and regular ward. In the ERAS clinic, nurse staff welcome patients who have made the decision for surgery and provide evaluation, education, and possible treatments. Other key members of the ERAS team include anesthesiologists, perfusionists, dietitians, and cardiac physiotherapy specialists. The main care elements are listed below, and the institutional implementation of ERAS can be found in Figure 1.

1. Preoperative Evaluation and Education: Data collection, laboratory analysis, education about the operative course, cessation of smoking and alcohol.
2. Anemia: Diagnosis and treatment by intravenous iron (5).
3. Optimization of blood glucose by checking of HbA1c and control of hyperglycemia.
4. Prehabilitation: Physical and pulmonary rehabilitation, nutritional support.
5. Psychiatric analysis: Alleviating anxiety.
6. Operative Period: Antibiotic prophylaxis, antifibrinolytic, acute normovolemic hemodilution, pain therapies, meticulous surgery by 3D endoscope, non-rib retraction, optimal cannulation site, goal-directed perfusion, less priming volume, normothermia, restrictive transfusion strategy.
7. Postoperative Care: Early extubation (within 6 h), prevention of nausea, aggressive analgesic treatment, oral intake, early mobilization (sitting outside of bed in the morning and walking in the evening of the first postoperative day), early removal of catheters and tubes (first postoperative day).
8. Early discharge after education and prescribing.

### Anesthetic management

In the operating theatre, patient is monitored by electrocardiogram, pulse oximetry, left radial artery cannulation. Following anesthesia induction with 2 µ kg^−1^ fentanyl, 2–3 mg kg^−1^ propofol, 1 mg kg^−1^ 2% lidocaine and 1.0 mg kg^−1^ rocuronium, intubation by double lumen endobronchial tube (Rüsch, Bronchopart®, France) via fiberoptic bronchoscopy is done. Arterial blood pressure, central venous pressure, Pleth Variability Index (PVI) and Perfusion Index (Masimo Root Platform, Masimo Corporation, Irvine, California, USA), esophageal temperature, and urine output were continuously monitored in addition to intraoperative transesophageal echocardiography (TEE). Anesthesia was maintained with 1%–2% sevoflurane in 50% oxygen—50% air and additional 1 µ kg^−1^ fentanyl boluses (total 5 μg kg^−1^) and 0.1–0.15 mg kg^−1^ rocuronium given for muscular relaxation as required. Depth of anesthesia was guided by bispectral index monitoring throughout the surgery. Noninvasive cerebral oxygenation values were continuously monitored via use of near-infrared spectroscopy (Masimo Root Platform, Masimo Corporation, Irvine, California, USA). According to patient blood management (PBM) protocols, acute normovolemic hemodilution and antifibrinolytic (tranexamic aside) is applied. All patients were given antiemetic prophylaxis (dexamethasone 4 mg), prophylaxis for gastric ulcer (ranitidin 50 mg), and antibiotics before skin incision. Noninvasive cerebral oxygenation values were continuously monitored via use of near-infrared spectroscopy (Masimo Root Platform, Masimo Corporation, Irvine, California, USA). Ventilator settings during one lung ventilation were tidal volume 4–6 ml kg^−1^, plateau airway pressure <20 cmH_2_O, peak inspiratory pressure <30 cmH_2_O, and positive end-expiratory pressure (PEEP) of 5–10 cm H_2_O (Dräger Perseus® A500, Lubeck, Germany). Arterial blood gases, electrolytes, hemoglobin, glucose, and lactate were monitored throughout the intraoperative period. Fluid replacement was established taking the need to avoid positive fluid balances into consideration while using crystalloids in line with the hemodynamic data and maintaining urine output above 1 ml kg^−1^ h^−1^. After completion of surgery, 1 g paracetamol was administered for postoperative analgesia and 4 mg ondansetron for nausea prophylaxis.

### Surgical setup

**Figure 2:**
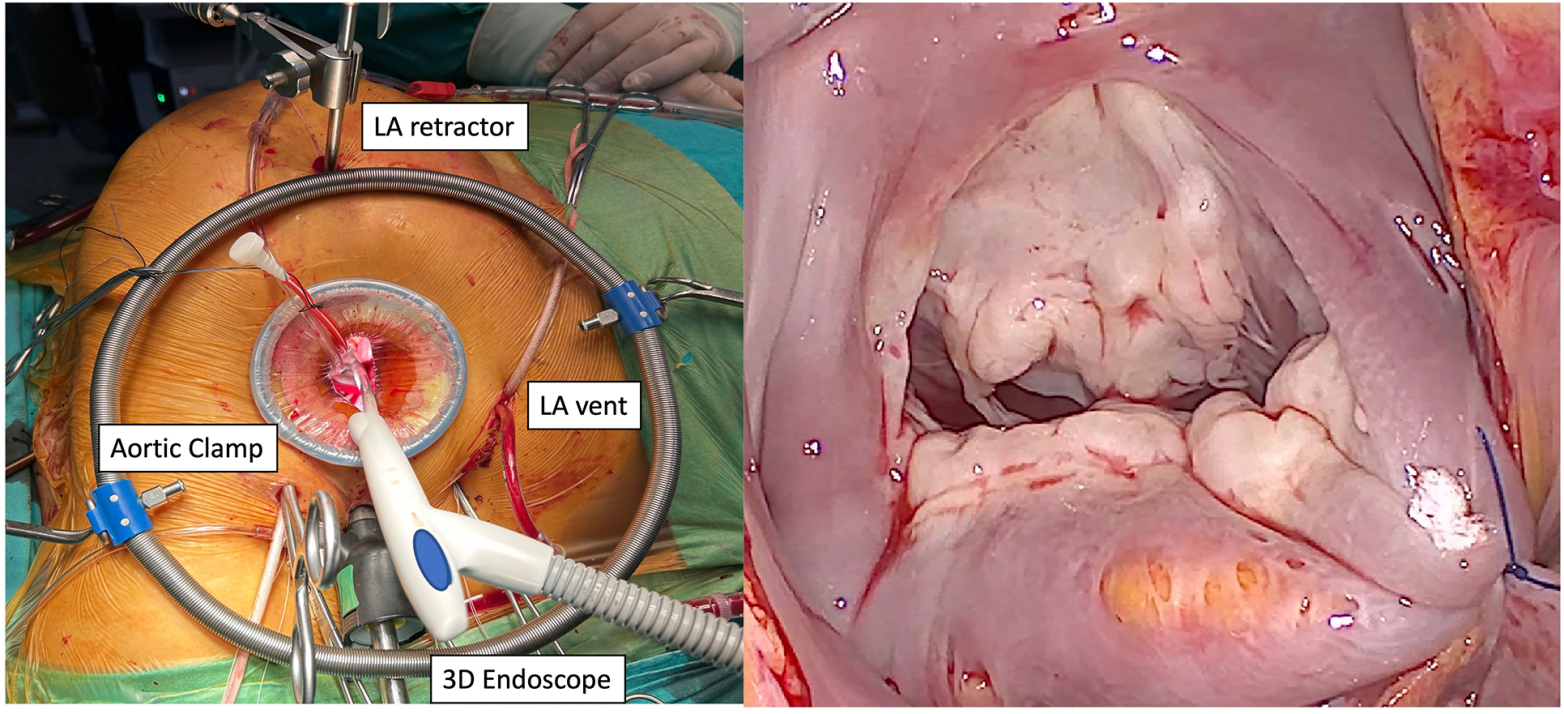
Intraoperative endoscopic setup and mitral valve exposure (LA, left atrium; 3D endoscope, 3-dimensional endoscope).

The surgical setup for TEMVS consisted of the small skin incision, surgical port with soft tissue retractor, and camera trocar from the 4th intercostal space (IS). Transthoracic aortic clamp was inserted from the 3rd IS and placed to the distal ascending aorta, and atrial vent cannula from the 6th IS through 7 mm flexible trocar (Figure 2). As a surgical preference, cardiopulmonary bypass (CPB) is initiated just before pericardiotomy and weaned off after closing the pericardium to provide safe surgical manipulation and optimize endoscopic view by deflating left lung. During surgery, multiple temporary wean from CPB is done for deairing, echocardiographic control and bleeding.

The data including preoperative (demographics, laboratory values, risk factors), intraoperative (surgical procedures, cardiopulmonary bypass, and aortic cross clamp times) and postoperative outcomes (the success of surgery, the amount of bleeding and transfusion, extubating time, the time of catheter removal, complications, length of postoperative ICU and hospital stay) are retrospectively analyzed in this study. As our program is in progress, some of the data (nutritional, psychologic and frailty status, rate of delirium and nausea, pain scores) are not recorded systematically. However, a comprehensive online data collection system including all ERAS protocols is developed by our team and will be used soon. The primary endpoints of the study are amount of bleeding and transfusion, postoperative complications, length of stay in the ICU and hospital.

### Statistical analysis

SPSS (IBM, version 25.0) was used for statistical evaluation, analysis and storage of the data obtained. Descriptive data were expressed in mean ± standard deviation (SD), median (min-max) or number and frequency, where applicable. Normally distributed continuous variables were expressed as mean ± standard deviation; for non-normally distributed variables, the median was used.

## Results

The data of 113 patients who underwent TEMVS have been analyzed. The mean age was 54.7 (±11.6) years and 56% of the patients were female. Most of the patients (54.8%) had type II dysfunction of the mitral valve, which is classified as degenerative in etiology. The rate of preoperative anemia (hemoglobin <13 g/dl) was 38.9%, and 49% of the patients had iron deficiency either with or without anemia. Thirty patients with iron deficiency anemia (IDA) were treated with intravenous iron replacement in our ERAS clinic. The other preoperative characteristics are presented in Table 1.

**Table 1:** Preoperative characteristics.

Preoperative characteristics	Number of patients (*n*: 113)	Value or percentage (%)
Age (mean, years)	n/a	54.7 (±11.6)
Gender (Female, percentage)	62	56%
Body Surface Area (mean, m^2^)	n/a	1.77 (±0.2)
Body Mass Index (mean, kg/m^2^)	n/a	25.2 (±4.2)
Hypertension	21	18.5%
Diabetes Mellitus	17	15%
Hyperlipidemia	20	17.6%
Preoperative Hemodialysis	2	1.7%
NYHA class		
Class I	20	17.6%
Class II	82	72.5%
Class III	11	9.9.%
Mitral Valve Dysfunction		
Type I	10	8.8%
Type II	62	54.8%
Type 3a	18	15.9%
Type 3b	23	20.3%
Left Ventricular Ejection Fraction (mean)	n/a	51.4% (±4.4)
Preoperative Atrial Fibrillation	38	33.6%
Hemoglobin (mean, g/dl)	13 (±2)	n/a
Anemia (Hb below 13 before treatment)	44	38.9%
Platelet count (mean, ×10^3^/µl)	n/a	248.8 (±89.2)
Ferritin (µg/L, sampled *n*: 105)		
Unchecked	8	7%
<30	22	19.4%
30–99	33	29.2%
>100	49	43.3%
Preoperative Iron Replacement	30	26.5%
Fibrinogen (mean, mg/dl)	n/a	308.07
CRP (mean, mg/L)	n/a	4.4 (±8)
Urea (mean, mg/dl)	n/a	34 (±10.9)
Creatinine (mean, mg/dl)	n/a	0.82 (±0.5)
GFR (ml/min/1.73 m^2^)		
<30	3	2.6%
30–60	9	7.9%
>60	111	89.5%
AST (mean, U/L)	n/a	19.9 (±8)
ALT (mean, U/L)	n/a	20.1 (±15.8)
Albumin (mean, g/L)	n/a	43.9 (±4.2)

NYHA, New York heart associatio; CRP, c-reactive protein; GFR, glomerular filtration rate; AST, aspartate aminotransferase; ALT, alanine aminotransferase.

The most frequent treatment was mitral valve repair (54.8%), with the success rate of 96.7% in degenerative group. Cryoablation was performed in 23.8% for any type of atrial fibrillation. The mean cardiopulmonary bypass (CPB) time was 149.9 ± 30.4 min, and the mean aortic cross clamp time was 94.8 ± 21.6 min. At the initial stage of the case series, Custodiol (histidine-tryptophan-ketoglutarate solution) was used for myocardial protection, while in the subsequent stage, Del Nido solution (a mixture of blood and crystalloid fluid in a 1:4 ratio) was preferred. The operative features are provided in Table 2.

**Table 2:** Intraoperative findings.

Intraoperative Findings	(*n*: 113)	Value or Percentage
Antifibrinolytics	70	61.9%
Acute Normovolemic Hemodilution	91	80.5%
Mitral Valve Treatment		
Repair	66	58.4%
Replacement	47	41.6%
Cryoablation	27	23.8%
Concomitant Tricuspid Annuloplasty	4	3.5%
Left atrial appendix closure	3	2.6%
Repair rate for type 2 mitral dysfunction	60/62	96.7%
Mean CPB time (min)	n/a	149.9 (±30.4)
Mean Aortic cross clamp time (min)	n/a	94.8 (±21.6)
Type of cardioplegia		
HTK-Custodiol	53	46.9%
Del-Nido (Blood)	60	53.1%
Type of venous cannulation		
Single femoral	90	79.6%
Jugular + femoral	23	20.4%
Type of arterial cannulation		
Femoral	113	100%

CPB, Cardiopulmonary Bypass.

In terms of clinical outcomes, the median amount of bleeding until the removal of the tubes was 250 ml. Five patients required re-exploration due to bleeding. Sixty-eight percent of patients did not require any red blood cell transfusion, while only 16% received a single unit. Fresh frozen plasma was not used in 90% of the patients, and platelets were not required in 99% of cases. The median length of stay in the ICU was 1 day, while the regular ward stay lasted for a median of 5 days. On the postoperative first day, 96% of foley catheters, 87% of all central venous catheter and 93% of all drainage tubes are removed. The rates of respiratory, infectious, and renal complications were 9%, 3.5%, 3.4% respectively. Mortality occurred in one patient (0.8%) who experienced postoperative bleeding and complications related to massive transfusion. The other postoperative complications are presented in Table 3.

**Table 3:** Postoperative findings.

Postoperative findings	Number (*n*: 113)	Value or percentage (%)
The amount of bleeding (median, ml))		250 ml
Extubation (median, hours)		7 h
Re-exploration due to bleeding	5	4.4%
Red packed cell transfusion		
None	77	68.1%
1 unit	18	15.9%
2 or more units	18	15.9%
Fresh frozen plasma transfusion		
None	102	90.2%
1 unit	5	4.4%
2 or more units	6	5.3%
Platelet Transfusion		
None	112	99.1%
Acute Kidney Injury		
Non-requiring HD	3	2.6%
Requiring HD	1	0.8%
New Onset atrial fibrillation	19	16.8%
Low cardiac output	9	7.9%
Respiratory complications	11	9%
Infective complications	4	3.5%
Wound complications	4	3.5%
Neurologic complications	0	0
Gastrointestinal complications	0	0
Sepsis	2	1.7%
Discharge Hemoglobin Level (mean, g/dl)	n/a	10.3 (±1.2)
Length of stay in ICU (median)	n/a	1 [1–18]
Length of stay in hospital (median)	n/a	5 [3–18]
Mortality	1	0.8%

ICU, Intensive Care Unit.

## Discussion

This study demonstrates the significant impact of ERAS protocols on patient outcomes in Totally Endoscopic Mitral Valve Surgery program. Our findings indicate early discharge (median of 5 days) with low rates of transfusion, complications, and mortality. These results, when compared with the latest database report of STS, seem to be better in terms of stroke, new onset atrial fibrillation, renal failure and operative mortality (6). Notably, the integration of TEMVS and multidisciplinary ERAS protocols, encompassing patient education, optimization of blood management, and postoperative care strategies, appears instrumental in achieving these positive outcomes.

The adoption of ERAS protocols should be regarded as a standard practice, with the potential to revolutionize patient care in cardiac surgery units worldwide through the utilization of a multidisciplinary approach (7). Surgeons and healthcare institutions may want to consider implementing similar protocols to improve patient recovery and optimize the use of resources. Furthermore, the use of digital systems to collect data specifically for the ERAS program will facilitate proper data storage and timely analysis, allowing for self-evaluation and the improvement of outcomes. Each component within the ERAS protocol plays a unique and essential role in influencing patient outcomes.

Through collaborative efforts, the ERAS team ensures a fair and efficient distribution of tasks and responsibilities. At this point, the structure of our ERAS team offers certain advantages compared to existing practices. In particular, nurses have been placed at the center of ERAS applications, with an emphasis on their active involvement from welcoming the patient in the preoperative clinic to discharge from the hospital. This approach has ensured the effective implementation of all steps in the process. Although the existing literature on nurse-centered ERAS team structures is quite limited, Ltaief et al. have provided a valuable report addressing the challenges related to education, assessment, and implementation within this framework (8).

In the following sections, we will discuss the most crucial elements of our program considering the latest evidence and with an eye towards future goals.

### Preoperative evaluation and education

Firstly, the ERAS clinic conducts an analysis of the demographics and risk factors, followed by the planning of appropriate treatment. One of the causes of anxiety among patients scheduled for surgery is the uncertainty surrounding the operative course. By implementing a structured evaluation and educational process that outlines each step of the surgical journey, patients are encouraged, the surgical team gains confidence, and the patient's anxiety is reduced. Additionally, this approach fosters greater collaboration from patients, particularly in relation to necessary treatments.

### Anemia

Anemia is associated with a notable increase in morbidity and mortality in cardiac surgery procedures (9). It was reported that one third of all patients was diagnosed with iron deficiency anemia (IDA) (10). Therefore, it is highly recommended to treat IDA before the surgery (11). In our clinic, following the protocol of Munoz et al, the patients with hemoglobin levels below 13 g/dl and ferritin levels below 100 or transferrin saturation rates below 25% receive treatment with ferric carboxymaltose (5). The operations of these patients are scheduled after a waiting period of 2 to 4 weeks. Unfortunately, due to reimbursement policies, the treatment of anemia other than IDA requiring Erythropoietin, is not available. Through this rigorous diagnostic and treatment regimen, nearly 70% of all patients were able to avoid transfusion.

### Prehabilitation

Patients who require cardiac surgery in the current era are older and more vulnerable, resulting in an increased risk of morbidity and mortality (12). Therefore, it is crucial to optimize their physiologic reserve to improve functional capacity after surgery and reduce the likelihood of complications. Prehabilitation, which consists of three components—worry, nutrition, and exercise, plays a significant role in this context (13). In our series, we did not select patients in a “frail” status for TEMVS during the learning curve period. However, according to our new protocol, patients will be evaluated for “frailty” and will be consulted by cardiac rehabilitation specialist for exercise. As we gain experience, we tend to prioritize older, frail, and more vulnerable patients for TEMVS. We believe that the combination of ERAS and TEMVS will be more beneficial to these “frail” patients.

### Totally endoscopic mitral valve surgery

Although all steps of the operative process are crucial for the comprehensive implementation of ERAS, meticulous surgery takes precedence. Following the TEMVS philosophy, every surgical intervention is minimized to reduce surgical trauma. It is essential to carefully select cannulation sites and cannula sizes to prevent perfusion complications. To minimize the risk of intervention, we primarily opt for single femoral venous cannulation and femoral artery cannulation. Despite the prolonged CPB times in our cohort, the optimal perfusion with vacuum assistance has resulted in a very low incidence of organ dysfunction and coagulopathy. Additionally, our institution has recently started utilizing goal-directed perfusion systems, a new technology that is recommended by recent guideline (4). In terms of surgical intervention, various intraoperative pain treatments such as cryotherapy, local perfusion catheters, and local neuroblockers are employed. However, the implementation of this care element is still a work in progress within our program.

### Patient blood management

The crucial role of patient blood management in the implementation of ERAS protocols in cardiac surgery is well-established by scientific evidence (14). In our program, blood-conserving techniques are employed through the collaborative efforts of anesthesiologists, perfusionists, and surgeons. When appropriate, antifibrinolytics supported by strong evidence and acute normovolemic hemodilution with moderate evidence are the preferred treatment options (15). By ensuring optimal perfusion with minimal hemodilution and implementing additional autologous priming techniques, we effectively prevent a decline in hemoglobin levels during cardiopulmonary bypass. The implementation of restrictive transfusion in whole perioperative period resulted in a transfusion rate of only 32% for red packed cells and 9% for fresh frozen plasma among the entire cohort. While five patients required re-exploration for bleeding during the early learning phase, the subsequent sixty cases exhibited no signs of bleeding.

### Postoperative care

In the initial phase of the intensive care unit, the team focused on goal-directed fluid therapy for the optimal utilization of vasopressors and fluids in managing hemodynamics. If appropriate, all patients were initiated for early weaning from mechanical ventilator (MV) to mitigate the occurrence of ventilator-induced lung damage and prolonged intubation-related anxiety. In our series, the median time for extubation was determined to be 7 h. Furthermore, the adoption of on-table extubation, a practice already employed by experienced centers and shown to enhance recovery, represents the next milestone for our ERAS team (16).

Concerning analgesic treatment, an escalating medical regimen (paracetamol, dextromethorphan, tramadol) is administered before and after extubation. Another aspect of our program that requires improvement is regional analgesic interventions by anesthesiologist.

The incidence of nausea in our series remained considerably low due to intraoperative prophylaxis and was managed through the administration of parenteral ondansetron. According to our developing protocol, patients will undergo an evaluation for the risk of nausea and subsequently receive treatment based on their level of risk (17). Regarding mobilization, nearly all patients were able to sit on chairs outside of their beds on the morning of the first day and ambulate under the supervision of nurses in the evening. We believe that the early removal of catheters, typically on the first day, enhances the patient's ability to mobilize and move freely, thereby contributing to a faster recovery and improved psychological well-being. Early discharge was a primary objective for our team if patients felt comfortable and confident both physically and mentally. The median length of stay was determined to be 5 days, with a few patients being discharged home on the 3rd postoperative day.

While our study provides valuable insights, it is not free from limitations. The sample size, although substantial, is limited to a single institution, which may affect the generalizability of the findings. Additionally, the retrospective nature of the study may introduce selection bias, and the collection of data for scoring systems and risk analysis are missing. Future studies with a larger, more diverse patient population, prospectively and comparative analysis would be beneficial in validating our results.

In conclusion, our study reinforces the notion that TEMVS, when combined with well-established ERAS protocols and team formation, leads to enhanced patient outcomes, including low rates of complication and transfusion, and expedited recovery. This integration marks a significant advancement in cardiac surgery, promoting not only patient well-being but also more efficient healthcare delivery.

## Data Availability

The raw data supporting the conclusions of this article will be made available by the authors, without undue reservation.
